# A Closer Look at Dystonia with the Glycosylation

**DOI:** 10.1007/s10571-025-01541-5

**Published:** 2025-03-18

**Authors:** Hours Camille, Gressens Pierre

**Affiliations:** Université Paris Cité, NeuroDiderot, Inserm, 48, Boulevard Sérurier, 75019 Paris, France

## Abstract

**Figure:**
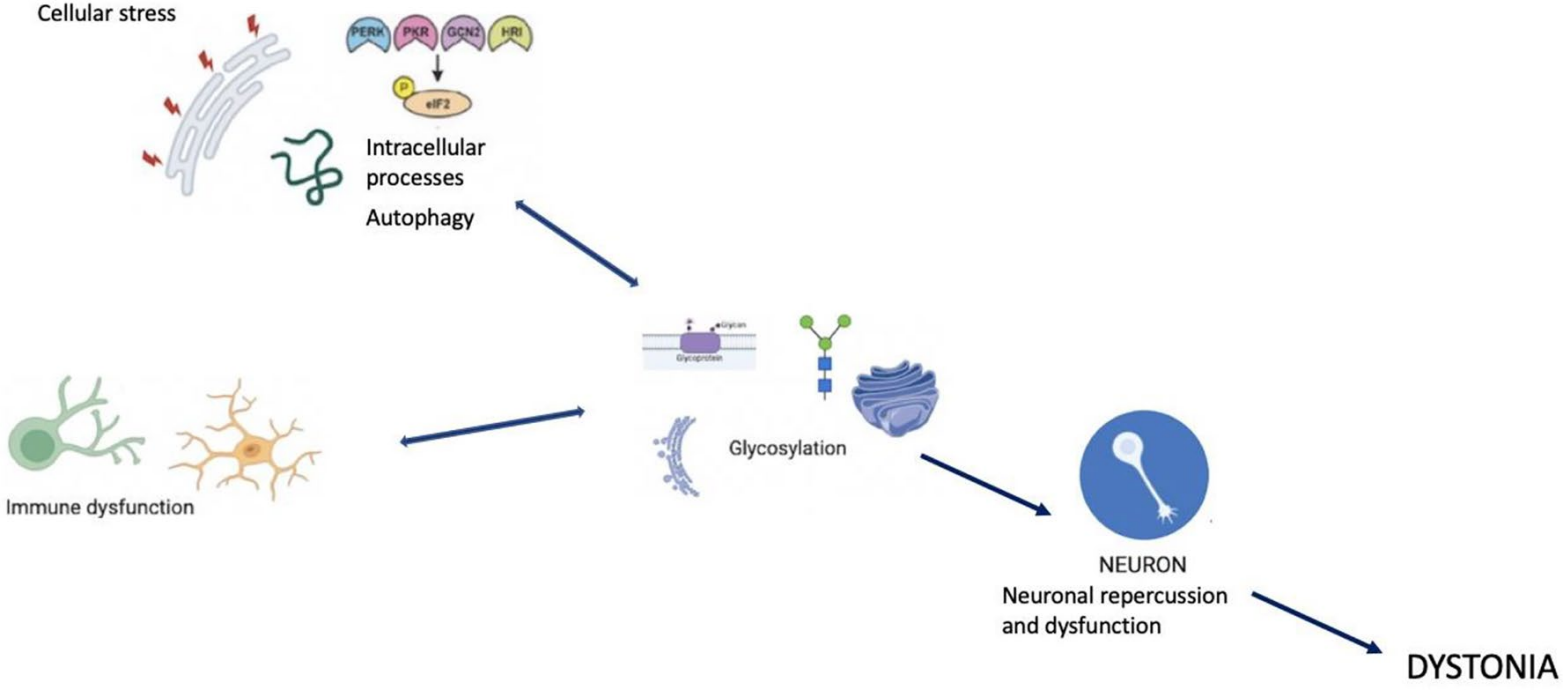
Glycosylation, Cellular Stress, and Immunity in Dystonia Pathogenesis. Glycosylation, cellular stress (eIF2α activation), and immune dysfunction converge to disrupt neuronal function and contribute to the pathogenesis of dystonia. Defective glycosylation can lead to oxidative stress, cellular apoptosis, and immune dysfunction, just as oxidative stress, cellular apoptosis, and immune dysfunction can lead to defective glycosylation. These processes, including ER stress and autophagy, interact with glycosylation and immune responses, leading to a better understanding of the molecular mechanisms underlying this neurodevelopmental disorder.

Glycosylation, Cellular Stress, and Immunity in Dystonia Pathogenesis. Glycosylation, cellular stress (eIF2α activation), and immune dysfunction converge to disrupt neuronal function and contribute to the pathogenesis of dystonia. Defective glycosylation can lead to oxidative stress, cellular apoptosis, and immune dysfunction, just as oxidative stress, cellular apoptosis, and immune dysfunction can lead to defective glycosylation. These processes, including ER stress and autophagy, interact with glycosylation and immune responses, leading to a better understanding of the molecular mechanisms underlying this neurodevelopmental disorder.

Dystonia is characterized by abnormally repetitive and involuntary movements or postures due to sustained and intermittent muscle contractions. It is classified according to its clinical features and underlying etiology. Dystonia may be genetic, idiopathic, acquired, or the result of other causes. It has a wide phenotypic spectrum and is frequently seen as part of complex neurological disorders. The term dystonia can describe an abnormal movement or refer to a group of diseases in which dystonia is the main feature, typically identified as a neurodevelopmental condition. Interestingly, dystonia is predominantly idiopathic, with most diagnostic tests being normal in dystonic patients, suggesting the presence of defects at the molecular level that are not identifiable by standard diagnostic tests. Genetic analysis can provide a final diagnostic for genetic forms (DYT forms). Dystonia is currently considered to be a brain network disorder resulting from abnormal neuronal motor programming (Stephen 2022). Consequently, understanding the pathophysiology of dystonia appears to be a challenge.

The pathogenic molecular pathways involved in dystonia are numerous, complex, and highly interconnected. This association of presumed mechanistic heterogeneity with syndromic presentation is found in other neurodevelopmental disorders such as Autism Spectrum Disorders (ASD). Despite the apparent molecular heterogeneity, there is a convergence towards a similar physiopathology. Some recurrent pathological functions have been identified in dystonia phenotypes, in particular, those related to gene transcription during neurodevelopment, calcium homeostasis, striatal dopaminergic signaling, endoplasmic reticulum (ER) stress response, and autophagy (Scarduzio and Standaert 2023). These neuronal dysfunctions impact synaptic function and plasticity, disrupt signaling pathways, and alter neurotransmitter synthesis and neuronal functions (Thomsen et al. 2024).

This major gap in the link between the different levels of analysis (genetic, biochemical abnormalities, circuit dysfunctions, behavior, and clinical manifestations) calls for a new perspective and a different approach to this issue. Synaptic function involving neuronal, glial, and immune cell types is regulated by a highly integrated mechanism, glycosylation with glycans functions. Proper glycosylation is essential for molecular function at different levels of the brain and is ultimately sensitive to changes in cellular states. Therefore, the study of extra-neuronal mechanisms, neuronal-glial interactions, and extracellular matrix function is a promising avenue (Yellajoshyula et al. 2022).

This perspective provides a new framework for reading the biological dysfunctions associated with dystonia:

1. Various biological processes are frequently described in dystonia, such as the cellular stress response with activation of aberrant EIF2*α* (eukaryotic initiation factor 2 alpha) signaling (a marker of the Integrated Stress Response (ISR)) in some forms of dystonia. Specifically, EIF2α is activated following various types of stressors, such as hypoxia and viral infections, leading to unfolded proteins in the ER (Beauvais et al. 2018). Dystonia due to genetic mutations (DYT forms) shows specific stress-focused abnormalities of the ER with functionally deficient secreted proteins (Thomsen et al. 2024). One of the central aspects and part of the final pathway in the pathogenesis of dystonia may be a dysfunctional post-translational pathway in the ER and dysfunction of nuclear transport. The ER and Golgi apparatus facilitate the addition of glycans to proteins and lipids, a crucial step in the morphology and function of neural stem cells during neurogenesis, particularly in embryonic development. Lysosomal function and autophagy also play roles in the pathophysiology of dystonia, with glycans playing a central role in autophagic responses, notably via Galectin3. Galectin3 senses damage-exposed glycans and coordinates the interaction between various molecular pathways for lysosomal repair (Jia et al. 2020). Dysfunctions in the cytoskeleton are another contributing factor in dystonia, as cytoskeletal proteins rely on proper glycoprotein conformation and functional glycosylation for attachment to the extracellular matrix. Cellular defects in the cytoskeleton are commonly found in various forms of dystonia (Beauvais et al. 2018).
2. Similar to ASD, although pathogenic variants in many genes have been identified in certain dystonic syndromes, the majority of patients do not benefit from a genetic diagnosis. Complex molecular processes may be at the start of the disease process. Research is taking interest in various forms of glycosylation, such as N- and O-glycosylation abnormalities, known to participate to neurodevelopmental disorders (Pradeep et al. 2023). However, little is known about their downstream targets. The modifications contributing to abnormal glycosylation are numerous and complex, occurring at different stages, from transcription to translation. They can result from mutations in the genes for glycosylation enzymes, abnormalities in the ER-Golgi complex, deficiencies in the availability of monosaccharides, and many other molecular processes necessary for the efficient functioning of glycoproteins. Hypoxia, one of the main causes of neurodevelopmental disorders, is closely linked to glycosylation. Their interaction has not yet been fully elucidated. Hypoxia induces the expression of glycosylation enzymes, leading to changes in cell function and migration, cell interactions with extracellular ligands, and signaling pathways. In the presence of cellular stresses, such as hypoxia and altered glycosylation, unfolded proteins accumulate, triggering proteolytic cleavage, nuclear transport and changes in gene expression. Post-translational histone modifications affect chromatin structure and function, leading to epigenetic changes. Defects in transcription regulation are a key pathogenic mechanism in dystonia and are also a strict regulator of epigenetic processes in the expression of glycosylation-related genes. These abnormalities have already been described in neurodegenerative diseases, autoimmune disorders, and cancer.
  For example, a specific type of O-glycosylation, O-GlcNAcylation (the addition of N-acetylglucosamine to serine/threonine residues of nuclear, mitochondrial, and cytoplasmic proteins) has been studied in neurodevelopmental disorders and congenital disorders of glycosylation (CDG) (Pradeep et al. 2023). CDGs are known to cause neurological disorders, particularly dystonia, and can be used as a model to study the neuronal and molecular consequences of glycosylation defects. CDG is associated with disorders of manganese metabolism which, independently of the diagnosis of CDG, is a frequent and well-established association with the phenotype of dystonia. The pathophysiological mechanisms identified in dystonia, such as oxidative stress, mitochondrial dysfunction, protein misfolding, ER stress, autophagy dysregulation and apoptosis, are similar to those observed in abnormalities of manganese metabolism. Manganese homeostasis and glycosylation are interdependent and have essential reciprocal effects (Foulquier and Legrand 2020). Unfortunately, there are currently no studies describing specific aspects of glycosylation in other forms of dystonia.
3. One of the main areas missing from dystonia research is immunity, which is directly linked to glycosylation. Glycosylation and immunity are very closely linked, since the state of glycosylation profoundly modifies the functions of immune cells in innate and adaptive systems (Radovani and Gugelj 2022). Interference between infectious events and the development of dystonia has been described, whether acutely during infection, subacutely or as a chronic complication, and glycosylation plays a central role in the infectious cycle by regulating the activation and evasion of the immune response (Pandey et al. 2022). One immune process that appears central and is currently best described in dystonia is autoimmunity: dystonia frequently co-occurs with dysimmune states such as autoimmune disorders (Dutta and Yadav 2024). Glycosylation is important for the development and function of B cells, which have both innate and adaptive immune activity. B cell is coated with glycosylated proteins and lectins that recognize glycosylated ligands on other cells. Glycans promote the development, maturation, and signal transduction of B cells via BCRs, the inhibitory co-receptor CD22, and Siglec-G. Changes in the glycosylation of B lymphocytes and their hyperactivation lead to the secretion of autoreactive antibodies, associated with changes in the glycosylation of IgG and changes in its structure, secretion and immune functions linked to autoimmunity. It should be noted that IgG secreted by plasma cells is the most abundant N-glycosylated protein in the human serum (Trzos et al. 2023).

However, there have been no detailed studies of immune changes in children with dystonia, particularly with regard to peripheral immune patterns and glial alterations.

A study on dystonia genes, particularly those associated with protein–protein interactions, revealed an over-representation of genetic pathways and ontologies involved in the immune system, transcription, metabolic pathways, endosomal–lysosomal mechanisms, and neurodevelopmental processes (Atasu et al. 2024). And glycosylation appears to be an underlying process at the origin of these dysregulations.

Therefore, although the molecular abnormalities observed in all forms of dystonia initially appear disconnected, they may be re-read to see their dependence on a ubiquitous, essential, and complex mechanism —glycosylation. Glycosylation is a central effector in the dynamics of the extracellular matrix, which ensures proper neuronal function.

## Data Availability

No datasets were generated or analysed during the current study.
